# Greater Heart Rate Responses to Acute Stress Are Associated with Better Post-Error Adjustment in Special Police Cadets

**DOI:** 10.1371/journal.pone.0159322

**Published:** 2016-07-18

**Authors:** Zhuxi Yao, Yi Yuan, Tony W. Buchanan, Kan Zhang, Liang Zhang, Jianhui Wu

**Affiliations:** 1 Key Laboratory of Behavioral Science, Institute of Psychology, Chinese Academy of Sciences, Beijing, China; 2 University of the Chinese Academy of Sciences, Beijing, China; 3 Special Police Academy, Beijing, China; 4 Department of Psychology, Saint Louis University, St. Louis, Missouri, United States of America; University of Zurich, SWITZERLAND

## Abstract

High-stress jobs require both appropriate physiological regulation and behavioral adjustment to meet the demands of emergencies. Here, we investigated the relationship between the autonomic stress response and behavioral adjustment after errors in special police cadets. Sixty-eight healthy male special police cadets were randomly assigned to perform a first-time walk on an aerial rope bridge to induce stress responses or a walk on a cushion on the ground serving as a control condition. Subsequently, the participants completed a Go/No-go task to assess behavioral adjustment after false alarm responses. Heart rate measurements and subjective reports confirmed that stress responses were successfully elicited by the aerial rope bridge task in the stress group. In addition, greater heart rate increases during the rope bridge task were positively correlated with post-error slowing and had a trend of negative correlation with post-error miss rate increase in the subsequent Go/No-go task. These results suggested that stronger autonomic stress responses are related to better post-error adjustment under acute stress in this highly selected population and demonstrate that, under certain conditions, individuals with high-stress jobs might show cognitive benefits from a stronger physiological stress response.

## Introduction

Errors are inevitable in everyday life and sometimes are even more likely to happen under conditions of stress. To adjust one’s own behavior after detecting an error to avoid more errors is critical in adapting to the environment, especially when facing stressors [1,2]. In real life, some populations, whose jobs are related to public safety, frequently meet high stress in their work, such as police officers, air traffic controllers, and firefighters. Such high-stress jobs require both an appropriate physiological response and behavioral adjustment to meet the demands of emergencies.

Behavioral adjustment after detecting an error has typically been observed in the form of longer response times for correct trials immediately following an error, which is referred to as post-error slowing (PES) [3,4]. The PES has been considered a behavioral adaptation that prevents future errors following the commission of errors [5]. The mechanisms underlying PES are increased recruitment and implementation of cognitive control [1,5,6] and/or attentional orientation to errors because of the infrequency of errors [7,8]. When PES mechanisms fail to adjust behavior after making an error, individuals may show compromised behavioral adjustment as reflected in lower post-error accuracy (double-errors) [9].

Physiological factors are associated with variation of PES. Previous studies found that PES is abolished after sleep deprivation or fatigue manipulation [10–12]. Besides, individuals with higher cardiorespiratory fitness [13], higher error-related autonomic nervous system activity [14], and higher pre-task cortisol levels [15] display greater PES. However, whether and how physiological responses to stress are related to PES is still unclear.

Stress causes a physiological reaction and has a variety of influences on the brain and behavior [16–18]. Physiologically, the immediate response of the autonomic nervous system to stress rapidly increases heart rate by exciting the cardiovascular system [18]. Furthermore, the response of the hypothalamic-pituitary-adrenocortical axis to stress can result in elevated circulating glucocorticoids [18]. These physiological responses are intimately correlated to behaviors under stress [16,17]. For example, previous studies showed that strong autonomic stress responses under challenging situations reflected psychophysiological effort [19], and cognitive functions including working memory and executive control were facilitated when individuals made efforts to actively cope with stress [16,20]. Regarding populations in high stress occupations, a recent study reported a positive relationship between physiological responses and cognitive functions among police officers. Officers with larger cortisol increases to an acute social stressor made fewer errors in a subsequent threat-related decision making task [21].

The purpose of the present study was to investigate the relationship between physiological responses to stress and behavioral adaptation following errors among a selected population with high-stress jobs. Therefore, special police cadets who were enrolled in special training for elite members of the police force were recruited for participation in the present study. Acute stress was induced by asking participants to perform a first-time walk on an aerial rope ladder bridge. As a standard part of cadet training, this stressor includes height exposure, which has been proven to be effective in inducing stress responses [22,23]. This stress condition was compared to a control condition in which participants walked on a cushion on the ground. The participants’ heart rate and subjective responses to the stressor or control task were measured. In addition, a post-stress/control Go/No-go task was used to investigate post-error behavior [24]. Based on previous studies demonstrating positive relationships between stress responses and cognitive functions especially among highly selected professionals [21], we hypothesized that the autonomic stress response, as reflected by increased heart rate, would be related to better post-error adjustment in special police cadets.

## Methods

### Participants

Sixty-eight male special police cadets, aged 21 to 27 years (mean = 23.98, SD = 1.13), participated in the present study. Because of the extremely low percentage of women among special police cadets, only male cadets were included. Due to the potential influence on stress responses, the following exclusion criteria were employed: 1) a cold or any medication use within two weeks of participation in the study; 2) chronic use of any psychiatric, neurological, or endocrine medication; 3) any major chronic disease; 4) any history of psychiatric or neurological disorders; 5) excessive alcohol consumption (more than two alcoholic drinks daily); and 6) chronic overnight work or an irregular day/night pattern. All participants were required not to 1) stay up late on the night before the study, 2) drink alcohol on the day of study, or 3) engage in vigorous exercise on the day of study. All participants had normal vision. None of the participants had any experience with the stressor used in the current study (see details below) until their participation. Participants were randomly assigned to the stress group or the control group. One participant in the stress group showed a No-go false alarm rate that was 3 SDs above the mean of all other participants’ false alarm rate, and one participant in the control group showed a hit response time (RT) that was 3 SDs above the mean of all other participants’ hit RT. These two participants were excluded from the analysis, resulting in 38 participants in the stress group and 28 participants in the control group. The mean ages (± SD) of the stress group and the control group were 24.00 (± 1.27) and 23.96 (± 0.92) years, respectively. There was no significant difference in age between these two groups (*t* (64) = 0.13, *p* > .05). All participants provided written informed consent prior to the study. This study was approved by the Ethics Committee of Human Experimentation of the Institute of Psychology, Chinese Academy of Sciences.

### General procedure

All testing took place in the afternoon from 14:30 to 18:30 in order to control for the circadian fluctuation of cognitive functions (e.g., [25]) and heart rate (e.g., [26]). Upon arrival, participants were given a brief introduction to the experiment in group sessions. Then, participants were tested in single sessions and not allowed to watch other participants performing the tasks. Pre-treatment heart rate (HR) data were recorded after participants had been seated and relaxed for 30 minutes, during which questionnaire data and basic demographic information were collected. Then, the participants were randomly assigned to the stress or control condition (see details below), during which HR data were continuously recorded. Subjective ratings of the treatment (see details below) were measured immediately after the treatment. Two minute after treatment participants completed a Go/No-go task (see details below) that lasted for approximately 14 minutes (14.17 ± 0.56 minutes). Post-treatment HR data were recorded upon completion of the cognitive task, that is, approximately 18 minutes (17.7 ± 1.5 minutes) after the end of the treatment.

### Stress induction and control condition

**Fig 1:**
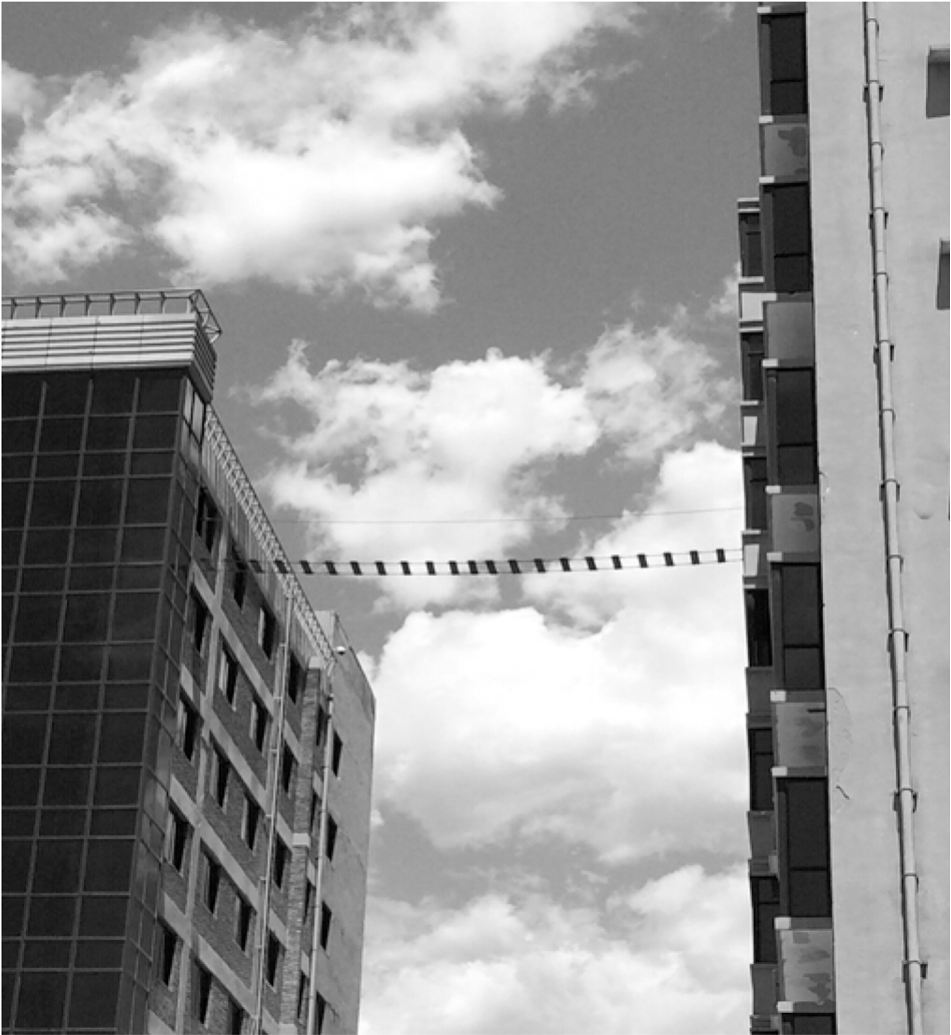
The Aerial Rope Ladder Bridge Used to Induce Stress.

To induce acute stress, the participants in the stress group were required to walk on an aerial rope ladder bridge. Although the chosen stressor is a standard part of cadet training, neither the stress group nor the control group had ever received such training before participating in the present study. The aerial rope ladder bridge was 18 meters in height and 12 meters in length (see Fig 1). Participants in the stress group were asked to walk two-thirds of the bridge and then turn around and walk back (16 meters in total). Upon turning around, they were asked to stand on the bridge and look down at the ground for 5 s (to increase stress intensity). It took the participants 65.3 (± 23.4) s, on average to complete the task. For safety, the participants wore a harness when walking on the bridge.

For the control condition, participants were asked to walk on a cushion on the floor for 16 meters in a straight line and to turn around at the midpoint. Thus, they walked for the same distance and in a similar way as the stress group on the aerial rope ladder bridge. The average time for the control group to complete the task was 58.8 (± 16.9) s.

### Go/No-go task

The settings and procedure of the Go/No-go task were identical to those of previous studies performed in our laboratory (e.g., [27]). The initial practice block consisted of 10 trials. After the practice block, two experimental blocks were completed, with 1–2 minutes breaks between them. Each experimental block consisted of 240 stimuli (20% No-go, 80% Go probability). The inter-stimulus intervals were randomly varied between 1200 and 1500 ms. For each trial, one of two letters (“O” or “X”) was presented in the center of the screen for 150 ms, with a visual angle of approximately 2.5° (vertical) and 2.2° (horizontal), and either a response (Go) or no response (No-go) was required. The participants were asked to respond as soon as possible in the Go trials by pressing a button on the keyboard with the index finger of their dominant hand. Two No-go trials were never presented consecutively. The association between the stimuli (“O” or “X”) and the Go or No-go response was counterbalanced across participants.

### Physiological and psychological measurements

The HR was recorded using a wireless chest HR transmitter and a wrist monitor recorder (Polar RSC800CX, Polar Electro, Finland). Pre-treatment HR data were recorded for two minutes when the participants were seated and had relaxed for 30 minutes. During treatment, HR data were continuously recorded throughout the stress or control treatment. Post-treatment HR data were recorded for two minutes after cognitive task completion. The HR was averaged across each measuring period using the Polar performance software and was defined as the number of beats per minute (bpm) [28,29].

Subjective ratings of the treatment were measured immediately after the stressor or the control task. The participants were asked to rate the following three questions on a 7-point scale: 1) how nervous were you when walking on the aerial rope ladder bridge/cushion (1 = *not nervous at all* and 7 = *extremely nervous*); 2) how fearful were you when walking on the aerial rope ladder bridge/cushion (1 = *not fearful at all* and 7 = *extremely fearful*); and 3) how much control did you feel when walking on the aerial rope ladder bridge/cushion (1 = *no control at all* and 7 = *in complete control*). Prior to the treatment, personality traits were assessed using the Big Five Personality Scale [30,31].

### Data analysis

To establish whether the induction of stress was effective, a 3 (Time: pre-treatment, during treatment, post-treatment) × 2 (Group: stress, control) repeated measures ANOVA was conducted for the HR data, and independent samples t-tests were conducted for the subjective measurements. For the behavioral data, the RT for hits, the miss rate in the Go trials, and the false alarm rate in the No-go trials were analyzed.

The present study focused on post-error behavior. The post-error trial refers to the Go trial following a false alarm trial. The post-correct trial, which refers to the Go trial following a hit trial, served as a control condition. Both RT and accuracy of the hit trials were calculated separately for the post-error and post-correct trials. The value of PES was calculated by subtracting the post-correct RT from the post-error RT. The post-error accuracy is reflected by the post-error miss rate increasing (PEMI) which was calculated by subtracting the post-correct miss rate from the post-error miss rate. The outliers (over three standard deviations from the group mean) were excluded respectively for PES and PEMI values.

Independent samples t-tests between the two groups were conducted on the post-error behavioral measures to examine the effect of acute stress on post-error behavior. Most importantly, Pearson’s correlation analysis between the HR increases (calculated by subtracting the pre-treatment HR from the HR during treatment) and PES/PEMI were performed for each group to examine the relationship between autonomic stress responses and post-error adjustment, as one-sample Kolmogorov-Smirnov tests showed that HR increase, PES, and PEMI were normally distributed (*p*s > .65). As education and personality traits may influence error control [32], these two factors were controlled in a partial correlation analysis. The reported *p* values are two-tailed. Bonferroni correction of significance level was used where multiple comparisons were made. The statistical analyses were performed using SPSS 18.0.

## Results

**Fig 2:**
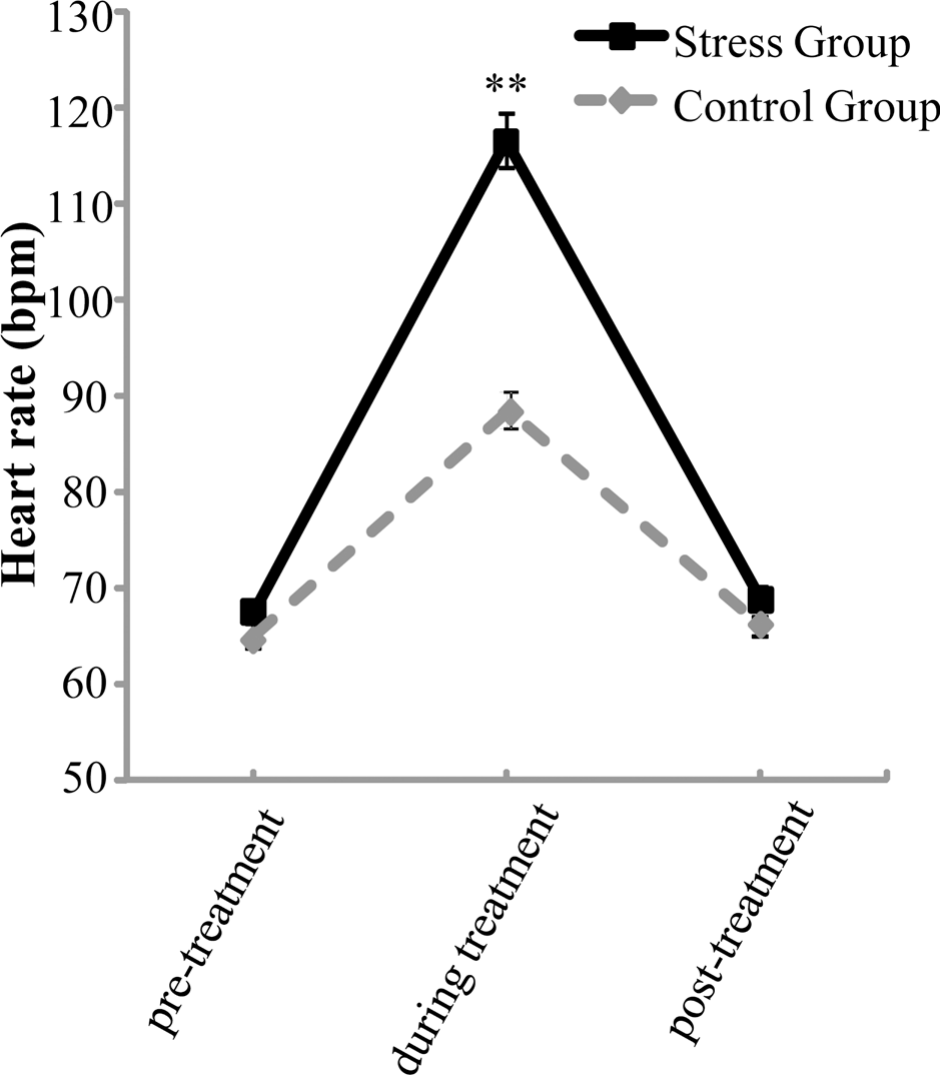
Mean Heart Rate Before, During, and After the Acute Stressor and Control Condition. Error bars are SEM (** *p* < .01).

Regarding HR, the repeated measures ANOVA revealed significant main effects of Time, *F*(2,128) = 484.323, *p* < .001, partial η^2^ = .883, and Group, *F*(1,64) = 29.763, *p* < .001, partial η^2^ = .317, and a significant Time × Group interaction, *F*(2,128) = 61.534, *p* < .001, partial η^2^ = .490. Post hoc analysis indicated a significantly higher HR during the treatment compared with both the pre-treatment and post-treatment measurements for both groups, *p*s < .001. Simple effect analysis revealed that the interaction was driven by the significantly higher HR of the stress group during the treatment compared with that of the control group (*p* < .001) (Fig 2).

For the subjective measurements (Table 1), independent samples *t*-tests revealed significantly higher ratings of nervousness, *t*(64) = 5.92, *p* < .001, and fear, *t*(64) = 5.32, *p* < .001, and a trend of lower “feeling of control” ratings in the stress group compared with the control group although the difference did not achieve significance after Bonferroni correction, *t*(64) = -2.03, *p* = .036.

**Table 1:** Descriptive Statistics for the Subjective Ratings of Acute Stress and Control Conditions.

Subjective measurements	Stress group		Control group		t value
	*M*	*SD*	*M*	*SD*	
Nervousness	3.11	1.35	1.36	0.91	5.92***
Fear	2.58	1.22	1.21	0.69	5.32***
Feeling of control	5.42	1.69	6.18	1.19	-2.03

M: mean; SD: standard deviation. Significant group differences are marked with asterisks. (*** *p* < .001)

Regarding the behavioral data, no significant group differences were found in the hit RT for Go trials (stress: 319 ± 49 ms; control: 311 ± 52 ms), the hit rate for Go trials (stress: 99% ± 1%; control: 99% ± 1%), or the false alarm rate for No-go trials (stress: 13% ± 11%; control: 19% ± 14%) (*p*s > .05).

For post-error behaviors, the number of post-error trials was 13 ± 11 for the stress group and 18 ± 14 for the control group. The PES for the stress group was 3.54 ± 56.56 ms (post-correct hit RT and post-error hit RT for the stress group were 323.80 ± 54.36 ms and 327.34 ± 80.45 ms, respectively, *p* > .05), and PES for the control group was -11.52 ± 50.60 ms (post-correct hit RT and post-error hit RT for the control group were 314.21 ± 55.31 ms and 302.70 ± 70.85 ms, respectively, *p* > .05). The PEMI for the stress group was 3.25% ± 11.72% (post-correct miss rate and post-error miss rate were 0.56% ± 1.00% and 3.81% ± 11.77%, respectively, *p* > .05), and the PEMI for the control group was 2.96% ± 8.09% (post-correct miss rate and post-error miss rate were 0.41% ± 0.64% and 3.37% ± 8.21%, respectively, *p* > .05). Neither PES nor PEMI differed between the two groups (*p*s > .05).

**Fig 3:**
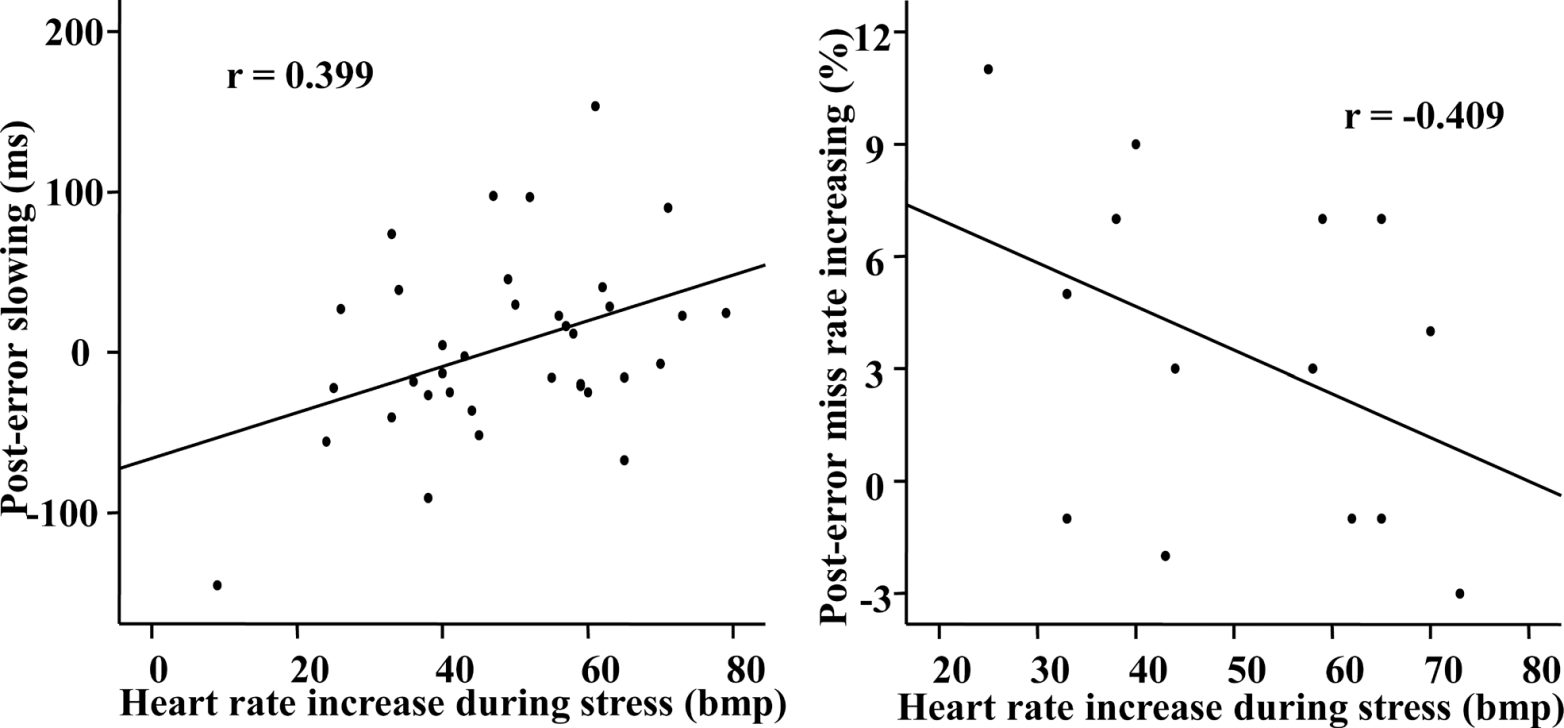
Scatter Plot of the Bivariate Correlation Between Heart Rate Increase During Stress and Post-Error Slowing (n = 36; *p* = .016, 2-tailed) (Left pannel) or Post-Error Miss Rate Increasing (PEMI) (n = 14, *p* = 0.146, 2-tailed) (Right pannel) in the Stress Group. Note that there were many ‘‘floor” values of PEMI (0% change) and we performed the correlation analysis without these floor values.

Regarding the relationship between autonomic stress response and post-error behaviors, the partial correlation analysis controlling for education and personality traits revealed a marginally significant (using Bonferroni corrected significance level) positive correlation between the HR increase during treatment and PES in the stress group, *r* = .391, *p* = .03 (Fig 3, left), but no such correlation was found in the control group, *r* = .072, *p* = .76. The difference between these two correlation coefficients found in the two groups was marginally significant (Z = 1.306, *p* < .10). The partial correlation analysis revealed a trend of negative correlation between the HR increase during treatment and PEMI in the stress group, *r* = -.495, *p* = .21, n = 14 (after excluding samples who showed no change in post-error accuracy and outliers over three standard deviations from the group mean) (Fig 3, right), but this partial correlation analysis cannot be conducted for the control group due to the limited remaining sample size (n = 7). Pearson’s correlation analysis showed that there were no significant correlations between the scores of the five personality subscales and HR increase/PES in both groups (*ps* > .05).

## Discussion

The aim of the present study was to investigate the relationship between physiological responses to acute stress and post-error adjustment in highly selected special police cadets. The height exposure stressor was effective for triggering an acute stress response, as demonstrated by the higher HR, higher subjective ratings of nervousness and fear in the stress group compared to the control group. Most importantly, the increase in HR during treatment was positively correlated with PES and had a trend of negative correlation with PEMI for the stress group. These results suggest that stronger autonomic stress responses are related to better post-error adjustment under acute stress in this highly selected population.

The HR response to stress is positively associated with PES and has a trend of negative association with PEMI. As increased PES and decreased PEMI have been considered better function in cognitive control following the commission of errors [1,5,6,9], the current results suggest that the special police cadets with higher HR increases during stress had better behavioral control after errors. This finding is consistent with previous studies showing that larger cortisol increases in response to an acute social stressor were associated with better performance in a subsequent decision-making task in police officers [21]. The results from the present and previous studies suggest that selected populations with high-stress jobs might show better cognitive functions with a stronger physiological stress response, which could be due to the intense training that habituates responses to stressful situations.

The mechanism underlying the positive association between HR increase during stress and behavioral control may be an increased recruitment of cognitive resources for individuals with higher HR increase under stress. Previous studies suggested that HR responses to stress reflect effort rather than distress (e.g., [19]). Such high levels of autonomic responses to stress are typically accompanied by effective performance because of increased mobilization of resources for high-level cognitive functions [16,18]. On the other hand, recruiting more resources for cognitive control according to experimental manipulation has been associated with better post-error behavioral adjustments [33]. Thus, participants with higher HR responses to stress in the present study may have mobilized more cognitive resources for behavioral adjustment following errors by increased cognitive control.

Another explanation may be that the mechanism that mediates the HR increase during stress may also mediate the behavioral adjustment following error trials. Previous studies found that individuals who exhibit better cardiorespiratory fitness showed larger increases in blood pressure during acute stress [34] as well as greater PES [13]. Accordingly, in the present study, greater HR increase during stress may reflect individuals’ better physical fitness, which may be the common mechanism underlying both greater acute stress response and better behavioral adjustment after making an error.

The HR increase during stress condition but not during the control condition was associated with PES, suggesting that psychogenic autonomic responses but not pure physiological autonomic activities are related to behavioral adjustment after errors. Previous studies revealed that both skin conductance responses during a task and pre-task cortisol levels were positively related to individuals’ PES [14,15]. The present study extends previous studies by showing that individuals’ autonomic responses to an acute stressor, as indexed by increased HR, are also predictive of their PES in subsequent tasks.

Our results revealed that no significant PES effect was shown at the group level. The literature on error processing suggests that PES is not always found. On the one hand, previous studies found that task parameters, such as the response-stimulus interval [35] and task instructions [36], affect the presence and magnitude of PES. For example, PES is reduced when task instructions emphasize response speed [36, 37, 38, 39]. In the present study, the instructions for the Go/No-go task also emphasized speed, which may have led to the diminished PES effect at the group level. On the other hand, PES has great individual variability. For the same task in a given sample of participants, PES may be large, weak, or even show a negative value [15, 40], which is the case in the current study. Notably, our results suggest that the levels of acute stress response are associated with individual differences in post-error behavior.

Several limitations of the present study have to be acknowledged. First, participants in the present study were special police cadets. Future studies should test whether the positive correlation between stress responses and cognition can be generalized to other highly selected professions. Second, as discussed above, better physical fitness may underlie a common mechanism for both greater HR responses to acute stress and better post-error adjustment. Therefore, future studies should include some index of physical fitness to test this hypothesis. Third, the stress intensity was relatively moderate in our study, as demonstrated by the relatively low subjective ratings of nervousness and fear (below the midpoint of the 7-point scale) and relatively high subjective ratings of the feeling of control (above the midpoint of the 7-point scale). It is possible that under greater stress intensity, the positive relationship between autonomic stress response and behavioral control would be reversed, even for these selected individuals with high-stress jobs, as several studies have revealed impaired cognitive function under stress (for a review, see [17]).

## Supporting Information

S1 File Yao et al., minimal data set. (SAV)
